# Impact of undernutrition and diabetes on the pharmacokinetics of first-line anti-TB drugs

**DOI:** 10.5588/ijtldopen.26.0084

**Published:** 2026-08-11

**Authors:** K. Ezhumalai, P.S. Rao, S.P. Babu, S.M. Jeyakumar, R.C. Bollinger, S.K. Heysell, J.A. Tornheim, P.B. Narasimhan, B. Thangakunam, M. Paradkar, D.J. Christopher, S. Gaikwad, V. Viswanathan, H. Kornfeld, J.J. Ellner, P. Salgame, C.R. Horsburgh, A.N. Gupte, A. Gupta, V. Mave, S. Sarkar, B. Perumal K, P. Sinha, on behalf of the RePORT India Consortium

**Affiliations:** 1 Department of Preventive & Social Medicine, Jawaharlal Institute of Postgraduate Medical Education and Research (JIPMER), Puducherry, India;; 2 Division of Infectious Diseases and International Health, University of Virginia, Charlottesville, VA, USA;; 3 Indian Council of Medical Research, National Institute for Research in Tuberculosis, Chennai, Tamil Nadu, India;; 4 Division of Infectious Diseases, Johns Hopkins University, School of Medicine, Baltimore, Maryland, USA;; 5 Department of Clinical Immunology, Jawaharlal Institute of Postgraduate Medical Education and Research (JIPMER), Puducherry, India;; 6 BJ Government Medical College and Sassoon General Hospitals, Pune, India;; 7 Christian Medical College, Vellore, Ranipet campus, Tamil Nadu, India;; 8 Center for Infectious Diseases in India, Johns Hopkins India, Pune, India;; 9 Byramjee Jeejeebhoy Government Medical College and Sassoon General Hospitals -Johns Hopkins University Clinical Research Site, Pune, Maharashtra, India;; 10 Prof. M. Viswanathan Diabetes Research Centre, Chennai, Tamil Nadu, India;; 11 Department of Medicine, University of Massachusetts Chan Medical School, Worcester, Massachusetts, USA;; 12 Department of Medicine, Rutgers New Jersey Medical School, Newark, NJ, USA;; 13 Department of Global Health, Boston University School of Public Health, Boston, MA, USA;; 14 Boston Medical Center, Section of Infectious Diseases, Boston, MA, USA;; 15 Boston University Chobanian and Avedisian School of Medicine, Section of Infectious Diseases, Boston, MA, USA;; 16 Department of Epidemiology, Boston University School of Public Health, Boston, MA, USA.

**Keywords:** tuberculosis, India, diabetes mellitus, body mass index, glycaemic status

Dear Editor,

Undernutrition and diabetes mellitus (DM) are common among people with TB (PWTB) in India and are each linked to unfavourable treatment outcomes.^1,2^ Low body mass index (BMI), a commonly used, if imperfect, assessment tool for undernutrition, and DM are each independently associated with reduced plasma concentrations of anti-TB drugs, potentially contributing to poor treatment outcomes.^3–5^ To understand individual and combined effects of undernutrition and DM on the pharmacokinetics (PK) of first-line TB drugs, we conducted a prospective, multicentre observational cohort study across five RePORT India consortium sites.^6^

We recruited adults (≥18 years) with microbiologically confirmed drug-susceptible pulmonary TB on first-line four-drug regimens administered via daily fixed-dose combination (FDC) tablets (Supplementary Data Figure S1). Participants were divided into four groups according to their nutritional and glycaemic status: normal weight and non-diabetic (N-N); underweight and non-diabetic (U-N); normal weight and diabetic (N-D); and underweight and diabetic (U-D). Underweight was defined as BMI <18.5 kg/m^2^; diabetes as self-reported history or baseline HbA1c ≥6.5%. We conducted PK sampling approximately 1 month after treatment initiation at pre-dose and 2, 4, 6, and 8 h post-dose. Plasma drug concentrations were quantified using validated high-performance liquid chromatography (HPLC).^7,8^ Population PK models were developed using nonlinear mixed-effects modelling in Phoenix WinNonlin 8.0 (Certara, USA) with first-order conditional estimation–extended least squares algorithm. We tested one- and two-compartment models with linear elimination, first-order absorption (with and without lag-time), and additive, proportional, and combined residual error structures. Covariates were evaluated by stepwise selection (forward inclusion *P* < 0.05, backward elimination *P* < 0.01). Final model selection used goodness-of-fit plots, objective function value, and Akaike and Schwarz information criteria (Supplementary Data Figure S2).^9^ Covariates included age, sex, BMI, serum creatinine, liver enzymes, HIV status, underweight status, and diabetes. We evaluated model robustness with visual predictive checks generated from 1,000 Monte Carlo simulations, which confirmed adequate prediction of the observed concentration-time data. We did not perform non-parametric bootstrapping as a separate diagnostic procedure. Final parameter estimates are in Supplementary Data Table S1. We used the final models to simulate peak concentrations (Cmax), and total interdose exposure by area under the concentration-time curve (AUC_0–24_) for individual participants. For rifampin, we simulated doses of 600, 900, and 1,200 mg; for isoniazid, 300 and 450 mg. We obtained ethical approvals at all research sites and obtained written consent from all participants.

Baseline characteristics and PK parameters for 127 participants with pulmonary TB are in Supplementary Data Table S2. Underweight participants with diabetes (U-D) had the lowest rifampin exposures. Underweight status was associated with significantly lower rifampin Cmax and AUC_0–24_ regardless of diabetes status. A one-compartment model with first-order absorption and lag-time best described rifampin; although underweight participants had lower rifampin exposure at the population level, no covariates met inclusion criteria for the final rifampin PK model. Only 38.6% of participants achieved the minimum rifampin Cmax target (≥8 mg/L) at actual doses (Figure panel A). Simulated flat dosing at 900 mg improved median Cmax to 11.1 mg/L and AUC_0–24_ to 71.2 mg·h/L; 1,200 mg further increased median Cmax to 14.8 mg/L (Supplementary Data Table S3). Relative to actual prescribing, flat 1,200 mg rifampin increased target attainment by 51.2% overall, 50.0% in those with diabetes, and 61.0% in underweight participants.

**Figure.**
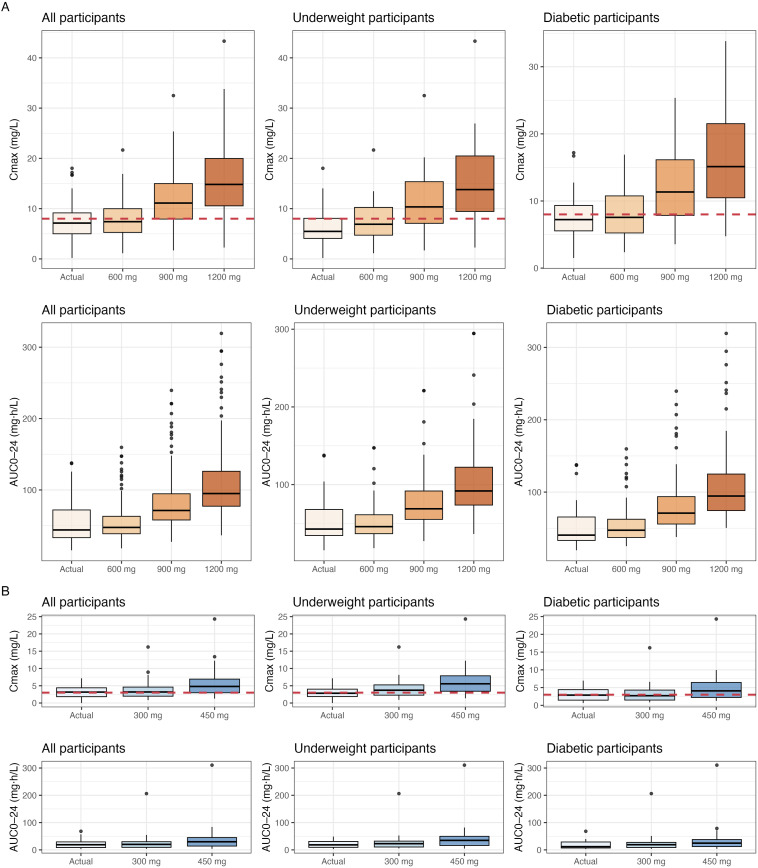
**A:** Simulated impact of higher rifampin doses on plasma exposure. Observed (‘Actual’) exposures at standard weight-based dosing are compared with model-based simulations at 600 mg, 900 mg, and 1,200 mg in all, underweight, and diabetic participants. **B:** Simulated impact of higher isoniazid doses on plasma exposure. Observed (‘Actual’) exposures at standard dosing are compared with model-based simulations at 300 mg and 450 mg in all, underweight, and diabetic participants. Boxplots show maximum concentration (Cmax, upper panels; dashed line represents the minimum Cmax target, defined as the lower bound of the accepted therapeutic range: ≥8 mg/L for rifampin [accepted range 8–24 mg/L] and ≥3 mg/L for isoniazid [accepted range 3–6 mg/L]) and AUC_0–24_ (lower panels). *P* values from paired comparisons between actual and simulated values.

Isoniazid Cmax and AUC_0–24_ did not differ significantly across nutritional or diabetes subgroups, and approximately 48% of participants fell below the minimum Cmax target (<3 mg/L). A two-compartment model with proportional error best fit isoniazid concentrations; BMI was a significant covariate for clearance and age for peripheral volume of distribution. Flat 450 mg dosing improved isoniazid Cmax in both underweight and diabetic participants, though AUC_0–24_ effects were more variable (Figure panel B; simulated median Cmax 4.79 mg/L, AUC_0–24_ 29.8 mg·h/L). Target Cmax attainment improved from 48.4% to 62.9% in participants with diabetes and from 45.8% to 83.1% in underweight participants.

Although previous studies have found an association between diabetes and the Cmax of isoniazid and pyrazinamide, our results did not corroborate this association.^4^ Pyrazinamide Cmax and AUC_0–24_ did not differ significantly across nutritional or diabetes groups. Given its high water solubility and efficient absorption, pyrazinamide has excellent bioavailability and is less likely to be influenced by host-related variability.^10^ Notably, weight-band FDC dosing appears to deliver adequate pyrazinamide and ethambutol exposure across all weight categories, with mg/kg doses remaining within or near WHO-recommended ranges throughout; by contrast, rifampin and isoniazid mg/kg fall toward the lower bound of recommended ranges at the upper end of each weight band, a pattern that may contribute to the subtherapeutic exposures observed in our cohort (Supplementary Data Figure S1).

In summary, underweight adults had substantially lower rifampin concentrations and reduced target attainment under current dosing, and higher doses improved simulated attainment. India’s weight-based algorithm prescribes lower absolute doses to underweight PWTB, suggesting that physiological impairment and systematic underdosing both contribute (Supplementary Data Figure S1).^11^ Although treatment outcomes were not directly assessed, subtherapeutic exposure may partly explain how undernutrition and diabetes undermine treatment effectiveness. Current fixed-dose regimens may compound this disadvantage. These findings argue for re-examination of rifampin dosing in underweight adults. Previous research has found a rifampin dose of 25 mg/kg to be safe among Indian PWTB.^12^ Prospective trials are trying to optimise dose (NCT06318416).

Several limitations warrant mention. The U-D group was the smallest subgroup and potentially limited detection of distinct PK patterns. HbA1c-defined diabetes at diagnosis may capture transient, disease-related hyperglycaemia rather than longstanding disease, introducing phenotypic misclassification. Ethambutol was not quantifiable by the HPLC-UV method we used, leaving the PK picture incomplete. Nearly all participants took medications with food, consistent with common practice among PWTB globally, which likely attenuated Cmax and prolonged Tmax.^13^ Finally, we did not genotype NAT2 acetylator status, leaving unmeasured variability in isoniazid PK.

The relationship between the subtherapeutic drug exposures observed here and adverse outcomes such as delayed culture conversion, treatment failure, and acquired drug resistance as well as treatment-related toxicity remains to be established prospectively in this population. This represents a critical gap and an important direction for future research. Within the existing programme infrastructure, supplementing the standard weight-band FDC with an additional rifampin tablet represents a pragmatic near-term strategy that warrants evaluation in prospective implementation studies. Whether nutritional support programmes or integrated diabetes management result in measurable improvements in drug exposure also represents an important and tractable question.

In conclusion, our results support three complementary strategies: routine assessment of nutritional status and glycaemic control at treatment initiation; re-examination of current weight-band dosing to avoid inadvertent rifampin underdosing; and consideration of therapeutic drug monitoring or empiric dose optimisation in individuals with poor early response.^14,15^ Aligning dosing strategies with host physiology represents a pragmatic, immediately actionable step toward more equitable and effective TB care.

## Supplementary Material
