# Supplementary material for: Impact of undernutrition and diabetes on the pharmacokinetics of first-line anti-TB drugs
Source: IJTLD Open. 2026 Aug 11;3(8):537–40. doi: 10.5588/ijtldopen.26.0084 (PMC13460429; doi:10.5588/ijtldopen.26.0084)
Supplement: Supplementary file 1 [file ijtldopen26-0084_supplementarydata1.pdf]

Supplementary Materials:

Supplementary Figure 1. Absolute and weight-adjusted doses of first-line anti-tuberculosis drugs under National Tuberculosis Elimination Programme (NTEP) fixed-dose combination weight-band dosing (intensive phase). Panel A shows absolute drug doses (mg) delivered by the NTEP 4-FDC (isoniazid 75 mg, rifampin 150 mg, pyrazinamide 400 mg, ethambutol 275 mg per tablet) across the continuous weight spectrum from 25–90 kg. Panel B shows the resulting mg/kg dose for each drug as a function of body weight, with shaded areas representing WHO-recommended target ranges (rifampin 8–12 mg/kg, isoniazid 4–6 mg/kg, pyrazinamide 20–30 mg/kg, ethambutol 15–20 mg/kg) and dotted vertical lines indicating weight-band boundaries.

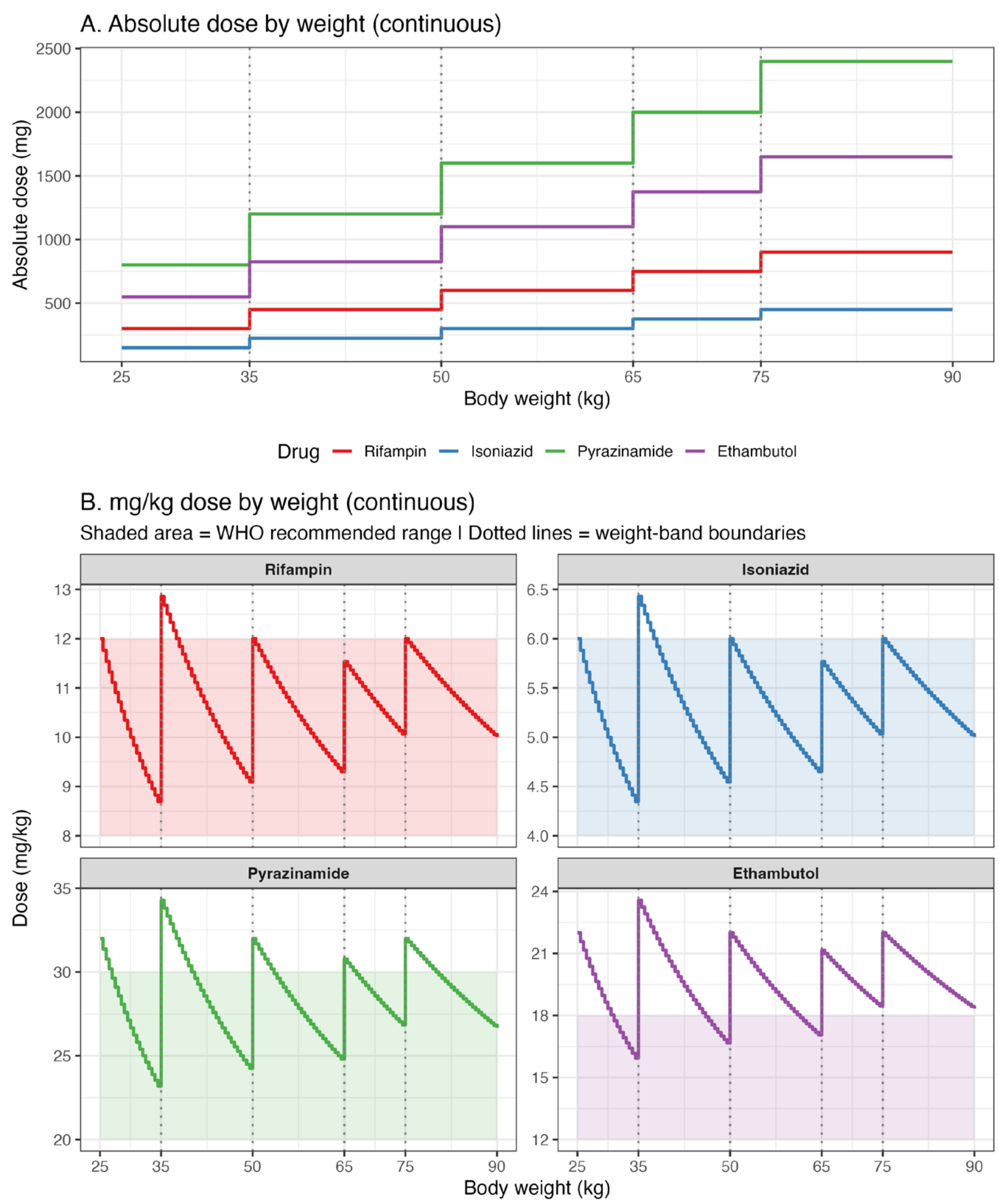

Supplementary Figure 2: Model diagnostic plots for rifampin and isoniazid.

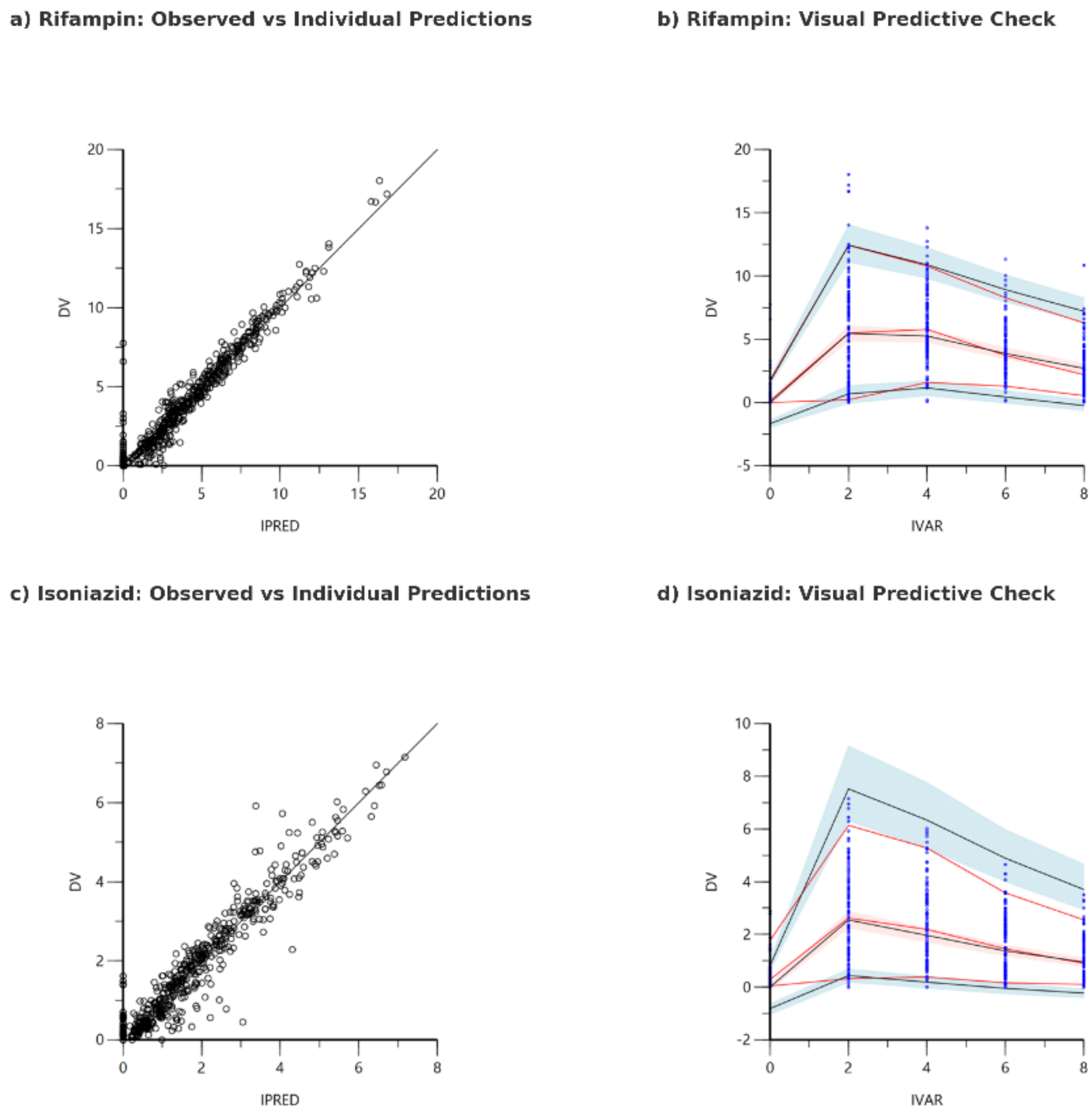

(a) Observed vs. individual predicted concentrations for rifampin. (b) Visual predictive check (VPC) for rifampin. (c) Observed vs. individual predicted concentrations for isoniazid. (d) VPC for isoniazid.

Supplementary Table 1: Final population pharmacokinetic parameter estimates for rifampin and isoniazid

Drug	Parameter	Estimate	%CV
Rifampin	Ka (1/h)	0.38	5.2
	V (L)	31.05	14.78
	Cl (L/h)	10.87	0.6
	Tlag (h)	0.14	18.42
Isoniazid	Ka (1/h)	1.29	1
	V (L)	64.5	8.38
	V2 (L)	130.69	46.72
	Cl (L/h)	9.28	24.72
	Cl2 (L/h)	3.17	41.48

Abbreviations: Ka, absorption rate constant; V, volume of distribution; Cl, clearance; Tlag, absorption lag time; V2, peripheral compartment volume; Cl2, intercompartmental clearance; CV, coefficient of variation. %CV represents the uncertainty of the estimated parameters. Covariate equation for the effect of body mass index, BMI, on clearance of isoniazid is given by: $Cl = 9.28 * BMI^{0.027}$ and the effect of age on peripheral compartment volume of isoniazid is given by: $V2 = 130.69 * Age^{0.099}$.

Supplementary Table 2: Baseline characteristics of groups and observed pharmacokinetic parameters

Characteristic	***N-D (n=41)***	***N-N (n=27)***	***U-D (n=21)***	***U-N (n=38)***	***p-value***
Age, mean (SD), years	46.07 (7.64)	43.30 (10.94)	45.71 (10.40)	36.13 (13.52)	<0.001
Male sex, n (%)	35 (85.4)	20 (74.1)	19 (90.5)	33 (86.8)	0.399
Ever smoker, n (%)	4 (9.8)	9 (33.3)	3 (14.3)	13 (34.2)	0.025
Alcohol use, n (%)	20 (48.8)	14 (51.9)	12 (57.1)	22 (57.9)	0.849
Living with HIV, n (%)	0 (0.0)	1 (3.7)	0 (0.0)	0 (0.0)	0.292
HbA1c, mean (SD), %	9.75 (1.90)	5.57 (0.45)	9.29 (2.56)	5.62 (0.41)	<0.001
BMI, mean (SD), kg/m²	22.77 (3.55)	21.08 (2.57)	16.39 (1.84)	16.50 (1.28)	<0.001
Albumin, mean (SD), g/dL	3.75 (0.43)	3.80 (0.51)	3.52 (0.51)	3.69 (0.52)	0.343
Rifampin					
C_{max}, mean (SD), μg/mL	8.45 (2.96)	8.11 (2.93)	6.19 (2.89)	6.27 (3.40)	0.003
t_{max}, mean (SD), h	3.02 (1.42)	3.19 (1.14)	4.10 (1.73)	3.32 (1.49)	0.051
AUC_{0-24}, mean (SD), μg·h/mL	40.57 (16.48)	40.90 (16.27)	28.22 (13.99)	28.23 (15.47)	<0.001
Subtherapeutic C_{max} n (%)	22 (53.7)	12 (44.4)	15 (71.4)	29 (76.3)	0.032

Isoniazid					
C_{max}, mean (SD), µg/mL	3.19 (1.73)	3.46 (1.58)	2.85 (1.74)	3.21 (1.57)	0.658
t_{max}, mean (SD), h	2.54 (1.00)	2.67 (1.11)	2.57 (0.93)	3.00 (1.59)	0.355
$AUC_{0\text{-}24}$, mean (SD), µg·h/mL	14.68 (9.40)	16.03 (9.29)	12.81 (8.30)	15.18 (8.07)	0.644
Subtherapeutic C_{max} n (%)	18 (43.9)	11 (40.7)	14 (66.7)	18 (47.4)	0.286
Pyrazinamide					
C_{max}, mean (SD), µg/mL	42.00 (15.75)	43.99 (16.88)	40.13 (8.57)	43.59 (13.87)	0.777
t_{max}, mean (SD), h	3.07 (1.49)	3.11 (1.50)	3.24 (1.18)	3.26 (1.64)	0.939
$AUC_{0\text{-}24}$, mean (SD), µg·h/mL	263.09 (109.59)	275.38 (110.29)	255.03 (57.52)	277.70 (94.63)	0.806
Subtherapeutic C_{max} n (%)	10 (24.4)	5 (18.5)	7 (33.3)	8 (21.1)	0.650
Number of drugs with subtherapeutic C_{max}					
0	11 (26.8%)	9 (33.3%)	3 (14.3%)	8 (21.1%)	0.434
1	13 (31.7%)	10 (37.0%)	6 (28.6%)	11 (28.9%)	
2	14 (34.1%)	6 (22.2%)	6 (28.6%)	13 (34.2%)	
3	3 (7.3%)	2 (7.4%)	6 (28.6%)	6 (15.8%)	

* N-N – Normal weight and non-diabetic; U-N – Underweight and non-diabetic; N-D – normal weight and diabetic and U-D – underweight and diabetic; p-values estimated using Kruskal-Wallis test.

Supplementary Table 3: Actual and simulated Cmax and AUC_{0-24} for rifampin and isoniazid

Subgroup	Dose	N	Cmax Median (IQR), mg/L	Subtherapeutic Cmax, n (%)	AUC_{0-24} Median (IQR), mg·h/L	Overall dose effect (p)
**Rifampin**						
All	Actual	127	7.13 (4.16)	78 (61.4%)	44.0 (38.8)*	<0.001 / <0.001
	Simulated 600 mg	127	7.41 (4.71)	69 (54.3%)	47.5 (24.5)	
	Simulated 900 mg	127	11.1 (7.07)	32 (25.2%)	71.2 (36.7)	
	Simulated 1200 mg	127	14.8 (9.42)	13 (10.2%)	95.0 (49.0)	
Diabetes	Actual	62	7.23 (3.78)	37 (59.7%)	40.5 (32.4)*	<0.001 / <0.001
	Simulated 600 mg	62	7.56 (5.52)	34 (54.8%)	47.3 (25.2)	
	Simulated 900 mg	62	11.3 (8.28)	16 (25.8%)	70.9 (37.8)	
	Simulated 1200 mg	62	15.1 (11.0)	6 (9.7%)	94.6 (50.5)	

Underweight	Actual	59	5.47 (4.00)	44 (74.6%)	42.8 (33.5)*	<0.001 / <0.001
	Simulated 600 mg	59	6.90 (5.52)	37 (62.7%)	45.9 (24.3)	
	Simulated 900 mg	59	10.3 (8.28)	19 (32.2%)	68.9 (36.5)	
	Simulated 1200 mg	59	13.8 (11.0)	8 (13.6%)	91.8 (48.7)	
**Isoniazid**						
All	Actual	127	3.16 (2.56)	61 (48.0%)	18.9 (20.9)*	<0.001 / 0.0003
	Simulated 300 mg	127	3.19 (2.63)	59 (46.5%)	20.2 (20.6)*	
	Simulated 450 mg	127	4.79 (3.95)	33 (26.0%)	29.8 (31.0)*	
Diabetes	Actual	62	2.89 (2.95)	32 (51.6%)	11.8 (22.0)*	<0.001 / NS
	Simulated 300 mg	62	2.72 (2.81)	33 (53.2%)	19.1 (18.9)*	
	Simulated 450 mg	62	4.08 (4.22)	23 (37.1%)	24.8 (25.8)*	
Underweight	Actual	59	2.83 (2.14)	32 (54.2%)	18.4 (21.5)*	<0.001 / 0.011
	Simulated 300 mg	59	3.71 (2.98)	26 (44.1%)	22.8 (21.5)*	
	Simulated 450 mg	59	5.56 (4.47)	10 (16.9%)	34.8 (33.4)*	

Notes: “Overall dose effect (p)” is ANOVA within subgroup reported as Cmax p / AUC p. Subtherapeutic Cmax defined as <8 mg/L for rifampin and <3 mg/L for isoniazid.
